# Fabrication of solderable intense pulsed light sintered hybrid copper for flexible conductive electrodes

**DOI:** 10.1038/s41598-021-94024-8

**Published:** 2021-07-15

**Authors:** Yong-Rae Jang, Robin Jeong, Hak-Sung Kim, Simon S. Park

**Affiliations:** 1 Department of Mechanical Engineering, Hanyang University, 222, Wangsimni-ro, Seongdong-gu, Seoul, 04763 Republic of Korea; 2 Department of Mechanical and Manufacturing Engineering, Schulich School of Engineering, University of Calgary, 2500 University Drive NW, Calgary, AB T2N 1N4 Canada; 3 Institute of Nano Science and Technology, Hanyang University, 222, Wangsimni-ro, Seongdong-gu, Seoul, 04763 Republic of Korea

**Keywords:** Nanoscale materials, Techniques and instrumentation, Chemical engineering, Mechanical engineering

## Abstract

Additively printed circuits provide advantages in reduced waste, rapid prototyping, and versatile flexible substrate choices relative to conventional circuit printing. Copper (Cu) based inks along with intense pulsed light (IPL) sintering can be used in additive circuit printing. However, IPL sintered Cu typically suffer from poor solderability due to high roughness and porosity. To address this, hybrid Cu ink which consists of Cu precursor/nanoparticle was formulated to seed Cu species and fill voids in the sintered structure. Nickel (Ni) electroplating was utilized to further improve surface solderability. Simulations were performed at various electroplating conditions and Cu cathode surface roughness using the multi-physics finite element method. By utilizing a mask during IPL sintering, conductivity was induced in exposed regions; this was utilized to achieve selective Ni-electroplating. Surface morphology and cross section analysis of the electrodes were observed through scanning electron microscopy and a 3D optical profilometer. Energy dispersive X-ray spectroscopy analysis was conducted to investigate changes in surface compositions. ASTM D3359 adhesion testing was performed to examine the adhesion between the electrode and substrate. Solder-electrode shear tests were investigated with a tensile tester to observe the shear strength between solder and electrodes. By utilizing Cu precursors and novel multifaceted approach of IPL sintering, a robust and solderable Ni electroplated conductive Cu printed electrode was achieved.

## Introduction

Recent advancements in flexible electronics have made way for exciting developments in devices such as: radio frequency identification (RFID) tags^1,2^, flexible displays^3–5^, flexible organic light-emitting diode (OLED)^6,7^, flexible solar cells^8,9^, and wearable electronics^10,11^. This is possible through use of flexible polymer-based substrates that can accommodate large deformations without sustaining structural damage. Utilizing common polymers as substrates such as polyimide (PI), polyethylene terephthalate (PET), and polyethylene terephthalate glycol (PETG) provide a pathway to a wide variety of robust and cheap electronic devices. However, these polymers are susceptible to damage from high temperature processes due to their low glass-transition temperatures ranging as low as 67 °C. As a result, modern high-temperature photolithography processes can be incompatible with flexible polymers without additional processing.

Alternatively, printing and IPL-sintering loose conductive nanoparticles can produce highly conductive tracks without thermal damage to the substrate. This low-temperature process can be streamlined to just three stages: printing-sintering-diagnosis. Metallic nanoparticles (NPs) are of interest in printed electronics due to their high electrical conductivity and simple ink-phase synthesis which can be readily implemented into existing printing technologies such as inkjet. Sintering via IPL bypasses high temperature processing by using the surface plasmonic effect; during IPL irradiation, electrons on the surface of nanomaterials resonate at a frequency that collapses its surface energy and allows significant necking/densification within the loose particles^12–14^. This effect significantly reduces sintering temperature and time requirements, which would otherwise damage the flexible substrate and immensely increase the manufacturing cost of flexible electronics. Due to the high surface area to volume ratio inherent in NPs, oxidation of metals is a large concern. Thus, noble and highly conductive metallic NPs such as gold (Au) and silver (Ag) are often used; however, these precious metals are prohibitively expensive, and their cost is further increased when processed into nanoparticle form. Copper (Cu) NPs are a lower cost option that still provides excellent electrical conductivity. To utilize Cu-based inks, Cu’s inherent susceptibility to oxidation must be addressed. IPL sintering has previously been utilized to reduce the Cu oxide shells by heat generation from surface plasmonic resonance and photo-degradation of polymeric binders^15^. IPL thus provides electrically conductive and oxide species reduction that reduce Cu particles for a high-performance ink receptive of existing printing technologies^16–18^.

Challenges still occur with IPL sintered Cu-inks in regard to their porosity, surface roughness, and mechanical strength. High porosity and surface roughness are of concern as it adversely affects solderability and thus inhibits integration with modern flexible printed circuit board (FPCB) manufacturing. Salam et al.^19^ confirmed that the poor solderability of printed Cu is due to its surface roughness and heterogeneity (mix of Cu and voids). In addition, polymeric binders are typically introduced to reduce copper oxides during IPL sintering. As polymeric binders thermally decompose, they leave behind pores which further increase the porosity issue of sintered nanoparticles^20^. Conventional sintering in powder metallurgy is done on previously pressed (‘green’) parts. These parts have already been compacted under high pressures which minimizes issues in final density and surface features. Compaction of printed-inks under high pressures, however, this adds further complexities and may damage the polymeric substrates. For these reasons, it has been difficult to realize the mass production of ink-printed electronic circuit components.

Electroplating is one method that can reduce surface roughness. Nickel (Ni) electroplating is an ideal candidate to provide a smooth, scratch-resistant, surface. A homogenous Ni surface facilitates good solderability while providing high mechanical strength without compromising electrical conductivity and oxidation susceptibility. Thus, a Ni plated printed Cu circuit produces a conductive, solderable (smooth surface), oxidation resistant, and mechanically resilient electrode^21–24^. However, Ni-electroplating deposition is limited to the surface and does not address internal porosity which can impair conductivity and adhesion to the polymeric substrate; in-addition, Ni deposition suffers from a lack of selectivity when presented with a conductive copper surface and is challenging to produce a desired circuit pattern. To address this, the initial non-conductive nature of unsintered inks can be utilized to form conductive and nonconductive patterns with selective exposure of IPL. By using a simple mask, an electrically conductive copper pattern is produced after IPL sintering; these patterns, specific to the electronic device or electrode requirements are sites of Ni electro-deposition while the non-conductive non-sintered regions do not participate in electroplating. This simple and selective plating of only IPL sintered tracks can be utilized to incorporate electroplating to the production of ink-based circuitry with minimal complexity.

In this study, solderability of printed and sintered Cu surfaces has been investigated by reducing internal porosity and surface roughness. To reduce internal porosity and surface roughness, Cu is seeded on Cu NPs. Chung et al.^25^ found that Cu precursors (Cu (II) nitrate trihydrate, Cu(NO_3_)_2_) exposed to IPL irradiation thermally degrade and precipitate as Cu (I) oxide. By introducing polyvinylpyrrolidone (PVP), these oxide Cu seeds are thermally degraded during IPL sintering to produce a dense and smooth Cu electrode. In addition, IPL is utilized to produce a circuit pattern that is selectively plated by Ni electroplating to enhance solderability and mechanical strength. The present novel application of multifaceted IPL sintering is optimized through initial ink composition as well as sintering and electroplating parameters. To assist in the parametric study of Ni electroplating, multi-physics FEM simulations were conducted; evolution of the Ni surface roughness as well as deposit thickness at various current densities, plating times, and bath temperatures were noted during simulations and experimentally verified.

The goal of the present work is to create a solderable electrode manufactured from printable Cu NP based ink. By utilizing IPL sintering with Cu precursors followed by Ni electroplating, a robust smooth electrode was produced. Experiments were performed to reveal additional characteristics of the present electrode production process such as: resistivity of the Cu precursor/NP (CPN) electrode using the 4-probe method; and surface morphology and cross-sectional analysis using both a 3D optical profiler and SEM after Ni electroplating of the IPL sintered CPN electrodes. Solder shear tests and simulations were conducted to investigate the correlation between the surface roughness and solderability. In addition, the contact angle of the solder was measured to confirm the wettability of solder on each type of electrode. Selectivity tests were performed by exposing only half of the printed electrode surface to flash-sintering. The half-electrodes were electroplated at optimal conditions to observe selective plating of sintered portions. Based on these results, the process conditions for solderable IPL sintered Cu electrodes were established for electrical circuit manufacturing by printed electronics technology.

## Experiments

**Figure 1:**
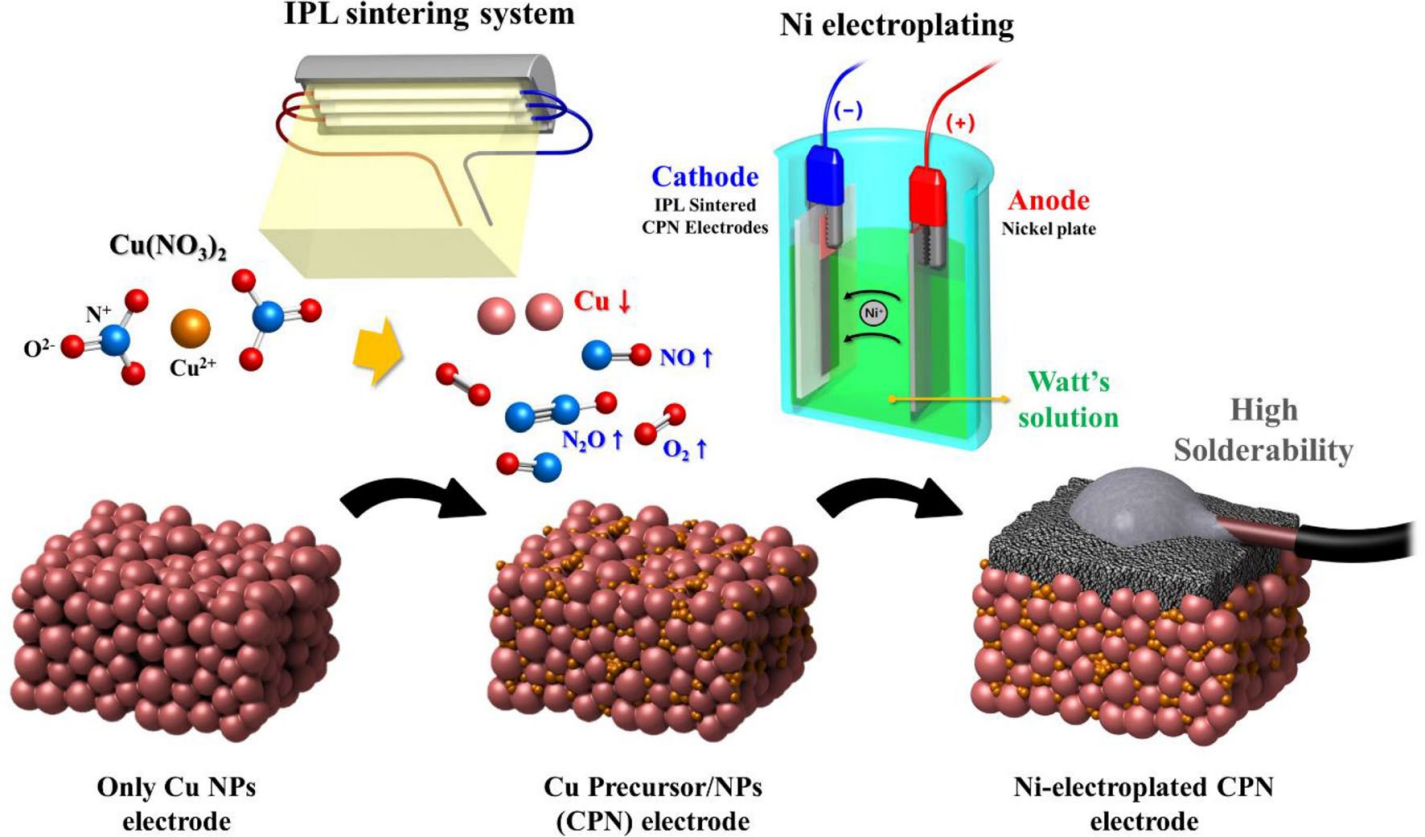
Highly solderable nickel electroplated Cu Precursor/NPs electrode.

A Cu-based conductive ink was fabricated by mixing the Cu NPs and Cu precursors. The Cu precursor/NP (CPN) ink was sintered with IPL irradiation followed by Ni electroplating (Fig. 1). Various properties of the Ni-electroplated CPN electrode were characterized to explore their effects on solderability.

### Fabrication of Cu NPs ink & Cu precursor/NPs (CPN) ink

For ink synthesis, poly(N-vinylpyrrolidone) (PVP, MW 55,000; Sigma Aldrich) (0.08 g) was dissolved in diethylene glycol (DEG, 99%; Sigma Aldrich) (1.08 g) and ultra-sonicated for 1 h. For Cu NPs ink, only Cu NPs (diameter, 50 nm) (2.16 g) were added to the PVP-DEG solution. For CPN ink, Cu NPs (diameter, 50 nm) (2.16 g), diethylene glycol butyl ether (DEGBE, 99%; Sigma Aldrich) (0.47 g), and the PVP-DEG complex solvent were vortexed for 1 min followed by 20 min of ultra-sonication. Cu precursors (Cu(NO_3_)_2_, 30 wt.% of Cu NPs; Sigma Aldrich) (0.648 g) were dispersed in a prepared solvent using an ultra-sonicator for 1 h (see Table 1).

**Table 1:** Material composition of Cu NPs ink and CPN ink.

Cu NPs ink composition		CPN ink compositions	
PVP	0.08 g	PVP	0.08 g
DEG	1.08 g	DEG	1.08 g
Cu NPs	2.16 g	DEGBE	0.47 g
		Cu NPs	2.16 g
		Cu(NO_3_)_2_	0.648 g (30 wt.% of Cu NPs)

**Figure 2:**
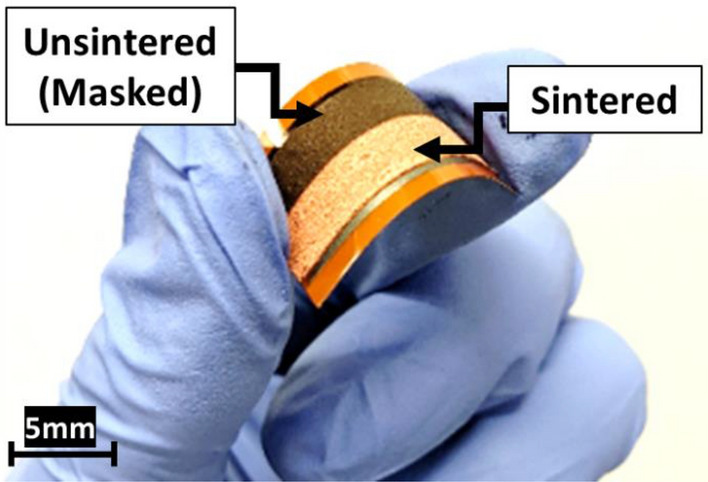
Half-sintered CPN ink flexible electrodes sintered at 2800 V for 3 ms (5.17 J/cm^2^) for selective electroplating investigations.

The CPN inks were printed using the doctor blade method using 50 μm thick polyimide (PI) substrates as 5 mm × 50 mm long tracks. The printed CPN ink was dried at 90 °C for 10 min on a hot plate followed by drying under vacuum for 12 h to completely remove residual solvents. IPL sintering was carried out with Xenon S-2300 Dual-Stage Sintering System with IPL irradiation conditions (pulse on-time, 3 ms; pulse number, 1; pulse energy, 3.04–5.88 J/cm^2^) to achieve a final sintered thickness of 25 μm. Samples prepared for selective electroplating were masked with aluminium foil to expose only half of the printed electrodes to IPL. By utilizing a simple mask (demonstrated here with aluminium foil), electroplating can be incorporated to produce a conductive, solderable, and mechanically robust printed circuit (Fig. 2).

### Nickel electroplating of CPN electrodes

A Watt's solution containing: nickel (II) chloride (NiCl_2_; Sigma Aldrich) (40 g/L), nickel (II) sulfate (NiSO_4_; Sigma Aldrich) (250 g/L) and boric acid (H_3_BO_3_; Sigma Aldrich) (45 g/L) was dispersed by sonicator for 24 hours^26,27^ (Table 2). For better electrical conductivity, semi-bright Watt’s solution was utilized. A nickel plate (2 cm × 8 cm), and IPL sintered CPN on the polymeric substrate were wired as the anode and cathode, relatively. 2, 5, 10 A/dm^2^ current densities were applied using a DC power supply (Model 9103; 42 V/ 20 A/ 320 W; BK Precision). In addition, for a smoother electroplated Ni layer, plating time (1, 3, 5 min) and bath temperature (40, 60 °C) were controlled. During the electroplating process, pH was fixed at 3.2 and the electrode distance between was fixed to 1 cm.

**Table 2:** Bath composition and operating conditions for semi-bright Watt’s solution Ni electroplating.

Bath composition		Operating conditions	
NiCl_2_	40 g/L	Current density	2, 5, 10 A/dm^2^
NiSO_4_	250 g/L	Time	1, 5, 10 min
H_3_BO_3_	45 g/L	Bath Temp	40, 60 °C
		pH	3.2
		Electrode distance	1 cm

A total of 18 electroplating conditions were explored and the surface roughness, thickness, and adhesion of the Ni layer on each electrode was measured. Surface roughness was measured using a 3D optical profiler (Zeta-20; Zeta Instruments). The thickness of the Ni layer was obtained by measuring the thickness of the CPN electrode prior to electroplating and then subtracted from the measured thickness after electroplating. For adhesion characterization, values from 0 to 5B were obtained through an ASTM D 3359 adhesion test. Following the ASTM D3359-B (the cross-cut tape test), each electrode was cut 6 times in both horizontal and vertical directions to form a total of 25 grids with sizes of 2 mm × 2 mm. It was cut by applying sufficient force so that it can be cut completely in the thickness direction of the electrode.

### Characterizations

The electrical resistivity of IPL sintered Cu NP, CPN, and electroplated CPN electrodes was obtained by converting the sheet resistance measured through the 4-point probe method. For Ni-electroplated CPN, the sheet resistance was measured in the form of a double layer in which a Ni layer was applied on an uneven Cu electrode. Therefore, the resistivity was calculated after assuming the entire Ni-electroplated CPN electrode as a metal alloy. The CPN electrode had a resistivity of 12.2 μΩ∙cm sintered under the optimum IPL conditions (pulse on-time, 3 ms; IPL energy density, 5.13 J/cm^2^), and the resistivity of the entire electrode considering each thickness of the Ni layer was calculated.

Among the 18 cases of Ni-electroplated CPN electrodes, the highest quality specimens (s-16) were used to compare with Cu NP and CPN (non-plated) electrodes. 3D optical profiler (Zeta-20; Zeta Instrument) was utilized to observe the surface roughness of each specimen. A root-mean-square deviation of surface topography (*S*_*q*_) was measured to compare surface roughness. The surface roughness analysis area was 831 μm × 623 μm with 200 height steps with a step size of 0.357 μm. Cross sectional analysis was performed to compare porosity and electroplating deposit characteristics. Each specimen was immersed in liquid nitrogen, frozen, and then broken to minimize deforming the cross-section of the specimen. The prepared specimens were subjected to SEM analysis to visually compare the packing density of each electrode layer. In the case of Ni electroplated CPN electrode, the distribution of surface components was identified through EDS analysis. To investigate the mechanical property of the Ni-electroplated CPN electrode as a FPCB, adhesion testing was conducted based on ASTM D3359.

### Soldering on the Ni-electroplated CPN electrodes

Solder (Sn99-Ag-0.3-Cu0.7) was applied to test the solderability of three different kinds of electrodes (i.e., Cu NPs electrode, CPN electrode, and Ni-electroplated CPN electrodes). The contact angle between the molten solder droplet and each type of electrode was measured to compare the effect of surface roughness to solder wetting using a contact angle measurement system (Smartdrop; Femtofab Co.).

In addition, shear strength between solder and the electrode was measured with a tensile tester (ESM303, 1.5 kN; Mark-10). Using solder wire and a soldering iron at 250 °C, a conductive wire was attached to the Ni-electroplated CPN electrode. The shear tensile test was performed by fixing the wire and PI substrate to the tensile tester after the solder was firmly hardened. The area between solder and electrode was kept to 20 mm^2^ in all cases, and the test was conducted at a constant speed of 0.3 mm/min. The tensile test proceeded until the solder was completely stripped from the electrode, and the recorded stress at failure was compared.

Through the above experiments, it was confirmed that the surface morphology of the electrodes is changed, and the solderability is improved through addition of Cu precursors and Ni-electroplating.

## Finite element method (FEM) simulation of Ni electroplating

Modelling for Ni electroplating on the CPN electrode and relationship between surface roughness and Ni-electroplating parameters are presented in this section. Ni-deposit behaviour was studied using a multi-physics FEM software by approximating the surface of the CPN electrode as a continuous row of triangles with fillets. To achieve these simulations, the electrodeposition tool in Chemical Engineering Module was selected. The deposition of Nickel layer on the Cu electrode is one of the modeling methods of the electrodeposition process in multi-physics FEM: ‘The standard model Copper Deposit in a Trench’. This simulation example is based on a research by Mattsson et al.^28^; where a rough copper electrode surface was applied to show how the Ni-electroplated surface developed depending on the cathode geometry as well as electrolyte composition.

**Figure 3:**
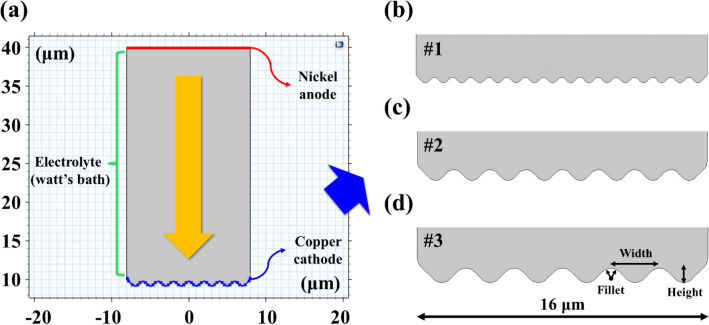
(**a**) The geometry of Ni electroplating model, and (**b**, **c**, **d**) the cathode electrode shape of 3 cases (dimensions are represented in Table 3).

To simulate the effect of initial cathode roughness on electroplating of Ni, the geometry of the CPN electrode was varied as shown in Fig. 3a. The anode was placed at the upper boundary while the cathode (with various roughnesses) was positioned at the lower boundary. To approximate surface roughness of sintered Cu particles the cathode was represented as a jigsaw shape composed of isosceles triangles. Furthermore, fillets were applied to the vertices of the aligned triangles (Fig. 3b–d). The dimensions of each case and RMS roughness are shown in Table 3.

**Table 3:** Geometry dimensions and RMS roughness of cathode electrode.

	#1	#2	#3
Triangle width (μm)	1.0	2.0	3.0
Triangle height (μm)	0.5	1.0	1.5
Fillet (μm)	0.25	0.5	0.75
RMS Roughness (μm)	0.097	0.194	0.394

Through this approximation, multi-physics FEM electroplating simulation was performed at various cathode geometries. As Ni was electroplated, the effectiveness of surface smoothening by electroplating was determined according to 3 cases of simulated CPN electrodes.

There are several governing equations involved in the simulation process^29,30^. First, the vertical walls on the main electrode are considered isolated:

1 $${\varvec{N}}_{i} \times n = 0$$

Second, the flow for each of the ions in the electrolyte is determined by the Nernst-Planck equation:

2 $${\varvec{N}}_{i} = - D_{i} \nabla c_{i} - z_{i} u_{i} Fc_{i} \nabla \phi_{l}$$

where ***N***_*i*_ is transport vector (mol/ (m^2^·s)), *c*_*i*_ is concentration in the electrolyte (mol/m^3^), *z*_*i*_ is the charge for the ionic species, *u*_*i*_ is the mobility of the charged species (m^2^/(s·J·mole)), $F$ is Faraday’s constant (As/mole), and ϕ_*l*_ is the potential in the electrolyte (V). This equation allows us to calculate the distribution of Ni ion concentrations, iso-potential lines, current density lines, and displacements of the cathode and anode surfaces.

In order to model the electrokinetics of the present Ni-electroplating simulation, the Butler-Volmer equation was used^31^. This equation represents the relationship of the current density of the electrode to the electrode potential with respect to the cathodic and anodic reaction and is given by:

3 $$i = i_{o} \left[ {\exp \left( { - \frac{{\alpha_{c} F_{\eta } }}{RT}} \right) - {\text{exp}}\left( { - \frac{{\alpha_{a} F_{\eta } }}{RT}} \right)} \right]$$

where $i_{o}$ is exchange current density, $\alpha_{c}$ and $\alpha_{a}$ are cathodic & anodic transfer coefficient, F is the Faraday’s constant, η is activation overpotential, and R is gas constant, respectively. The electrolyte initial condition and parameters of Ni electroplating model are in Table 4.

**Table 4:** The electrolyte initial condition and parameters of Ni electroplating model.

Parameter	Value	Description
E_eq__Ni	− 0.26 (V)	Equilibrium potential, nickel reaction
Kappa	10 (S/m)	Electrolyte conductivity
M_Ni	58.69 (g/mole)	Molar mass, nickel
M_Cu	63.55 (g/mole)	Molar mass, copper
Rho_Ni	8908 (kg/m^3^)	Density, nickel
Rho_Cu	8960 (kg/m^3^)	Density, copper
i_o__Ni	0.1 (A/m^2^)	Exchange current density, nickel reaction
i_o__H	2E-5 (A/m^2^)	Exchange current density, hydrogen reaction

Thus, the thickness and shape of the electroplated Ni layer can be determined through simulations with respect to time. Therefore, the deposition and smoothening process of Ni layer can be predicted over time at various conditions of Ni-electroplating (ex. current densities and temperatures). By utilizing simulated results, optimal Ni-electroplating parameters were predicted prior to experimentation.

## Results and discussions

The evolution of surface roughness during Ni electroplating was optimized experimentally and compared to simulation results. By optimizing the electroplating parameters for the smoothest surface, solder adhesion is also expected to be optimized. In addition, various flash energies and their effect on the sintered structure was characterized. The effect of ink compositions and electroplated samples was investigated to provide insight of an individual components influence on final performance. Finally, a high-performance flexible printed track was produced with high solderability.

### IPL sintering of the Cu NPs/precursor electrodes

**Figure 4:**
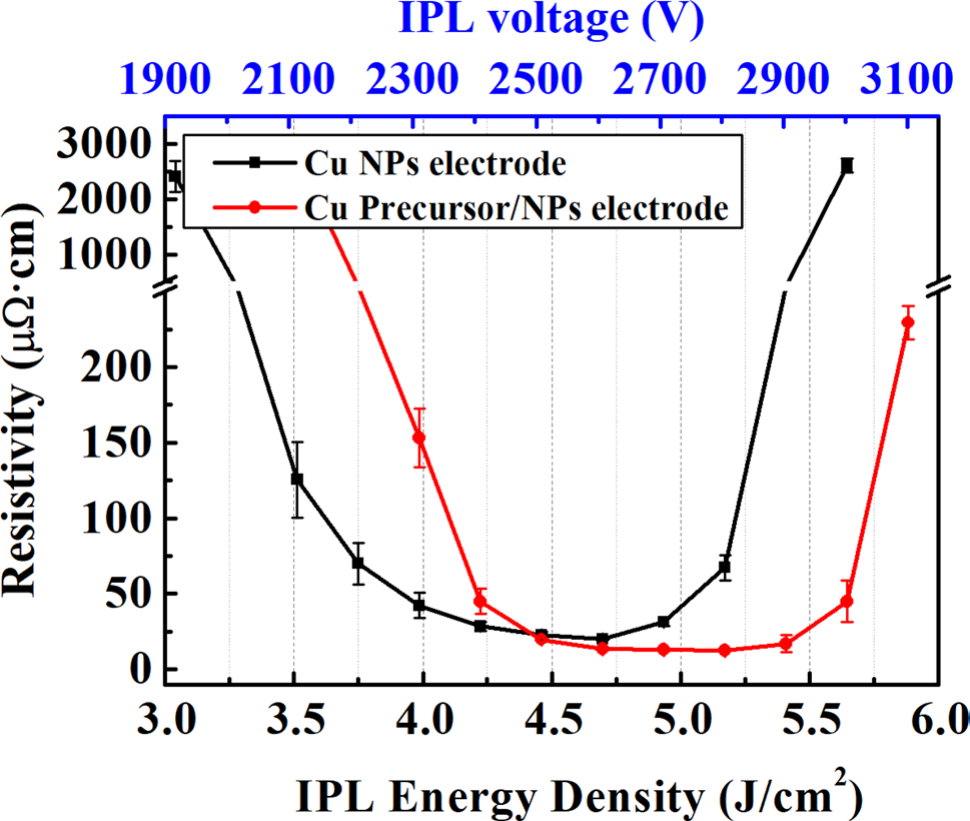
A plot of resistivity changes in Cu and CPN electrodes with respect to applied IPL irradiation energy.

From previous research, it was found that Cu precursor in an amount of 30 wt.% relative to Cu NPs has the highest electrical conductivity and packing density when sintered with IPL^25^. In accordance to the previous findings, 30 wt.% of Cu precursor is utilized in this study to improve packing density and electron pathway. The specimens were sintered with various IPL irradiation energies as shown in Fig. 4 and Table 5. The resistivity of the Cu NPs electrode and CPN electrode was the lowest when treated with an irradiation energy of 4.70 J/cm^2^ and 5.13 J/cm^2^, respectively. It was found that the CPN electrode had lower resistivity when the IPL energy density was over 4.46 J/cm^2^. However, when energy densities above 4.94 J/cm^2^ and 5.41 J/cm^2^ were applied to Cu NP and CPN respectively, resistivity began to increase. This was the result of oxidation on the copper electrode that formed from an excessive increase in temperature. Overall, the optimal IPL energy densities of 4.70 J/cm^2^ and 5.17 J/cm^2^ was selected for Cu NPs and CPN electrodes, respectively based on the lowest resistivity readings.

**Table 5:** IPL energy density with respect to IPL voltage at 3 ms pulse on-time and resistivity of Cu NPs electrode and CPN electrode for each IPL energy density.

IPL voltage (V)	IPL energy density (J/cm^2^)	Resistivity of Cu NPs electrode (μΩ∙cm)	Resistivity of CPN electrode (μΩ∙cm)
1900	3.04	2406.21 (± 277.78)	–
2000	3.28	431.10 (± 58.33)	–
2100	3.51	125.54 (± 25.12)	2783.88 (± 297.78)
2200	3.75	69.99 (± 13.89)	339.43 (± 52.78)
2300	3.99	42.21 (± 8.33)	153.32 (± 19.44)
2400	4.22	28.32 (± 2.78)	44.99 (± 8.33)
2500	4.46	22.77 (± 1.94)	19.71 (± 1.71)
2600	4.70	19.98 (± 0.56)	13.60 (± 1.11)
2700	4.94	31.10 (± 2.78)	12.93 (± 0.83)
2800	5.17	67.21 (± 8.33)	12.20 (± 0.28)
2900	5.41	347.77 (± 53.83)	16.93 (± 5.56)
3000	5.65	2606.32 (± 127.78)	44.99 (± 13.89)
3100	5.88	–	229.43 (± 11.11)

The lowest resistivity of 19.98 μΩ∙cm was observed at the optimum IPL energy density (4.70 J/cm^2^) for the Cu NP electrode. On the other hand, the optimum IPL energy of CPN electrodes was found to be higher than that of Cu NP electrodes. This was due to a higher energy requirement for thermal decomposition of the Cu precursor. The CPN electrode showed the lowest resistivity of 12.2 μΩ∙cm at its optimal IPL energy density (5.17 J/cm^2^). This indicated that the voids between Cu particles were filled with Cu seed precipitated from thermal decomposition of Cu precursor. The thermal decomposition of Cu precursor and reduction of oxides is presented in the following equations:

4 $${\text{4Cu}}\left( {{\text{NO}} _{{3}} } \right)_{{2}} \to {\text{4CuO}} + {\text{4NO}} \uparrow + {\text{ 2N}}_{{2}} {\text{O}} \uparrow + {\text{ 7O}}_{{2}} \uparrow$$

5 $${\text{5CuO}} + {\text{CH}}_{{3}} {\text{COOH}} \to {\text{3Cu}} + {\text{Cu}}_{{2}} {\text{O}} + {\text{2H}}_{{2}} {\text{O}} + {\text{2CO}}_{{2}} \uparrow$$

6 $${\text{4Cu}}_{{2}} {\text{O}} + {\text{CH}}_{{3}} {\text{COOH}} \to {\text{8Cu}} + {\text{2H}}_{{2}} {\text{O}} + {\text{2CO}}_{{2}} \uparrow$$

Cu^2+^ ion in Cu (II) nitrate trihydrate was reduced to Cu (I) oxide and Cu (II) oxide step by step (Eq. 4–6)^32,33^. It is well documented that the reduction of Cu oxide proceeds by photo-thermal degradation of PVP binders^15^. In summary, Cu precursor is thermally decomposed by the heat generated by surface plasmonic resonance of Cu NPs by IPL irradiation and then formed into Cu seeds by photo-thermal degradation of PVP binder.

Through this process, CPN electrodes achieved higher electrical conductivity and packing density than Cu NP electrodes. Sintering CPN electrodes under optimal conditions significantly reduced internal voids, surface roughness, and achieved homogeneous surface characteristics. However, the CPN electrodes still had unsatisfactory mechanical and solder wetting properties. Therefore, Ni electroplating was performed to further lower the surface roughness of the electrode and improve its mechanical properties and solderability.

### Nickel electroplating

For improved soldering on printed Cu electrodes, surface roughness and internal voids should be minimized to create a homogeneous surface. The use of Cu precursors helped fill the internal voids in the Cu NP electrodes and helped to decrease the surface roughness compared to when only Cu NPs were used^25^. The improvement of packing density and surface roughness by addition of Cu precursor is specified in Fig. 7 and Table 9 for comparison with Ni-electroplated CPN electrode.

To further improve surface properties, Ni-electroplating was utilized. Prior to the experiment, FEM simulation was performed to predict the degree of smoothening of the CPN electrode at various Ni electroplating conditions.

FEM simulation was conducted with an important characteristic/assumptions: the shape of the Cu electrode was approximated as shown in Fig. 3. The electroplating time required for the simulated Ni deposit surface to reach an RMS value below 0.01 μm is taken as the time required for successful smoothening of the electrode surface, or *flattened time*.

**Figure 5:**
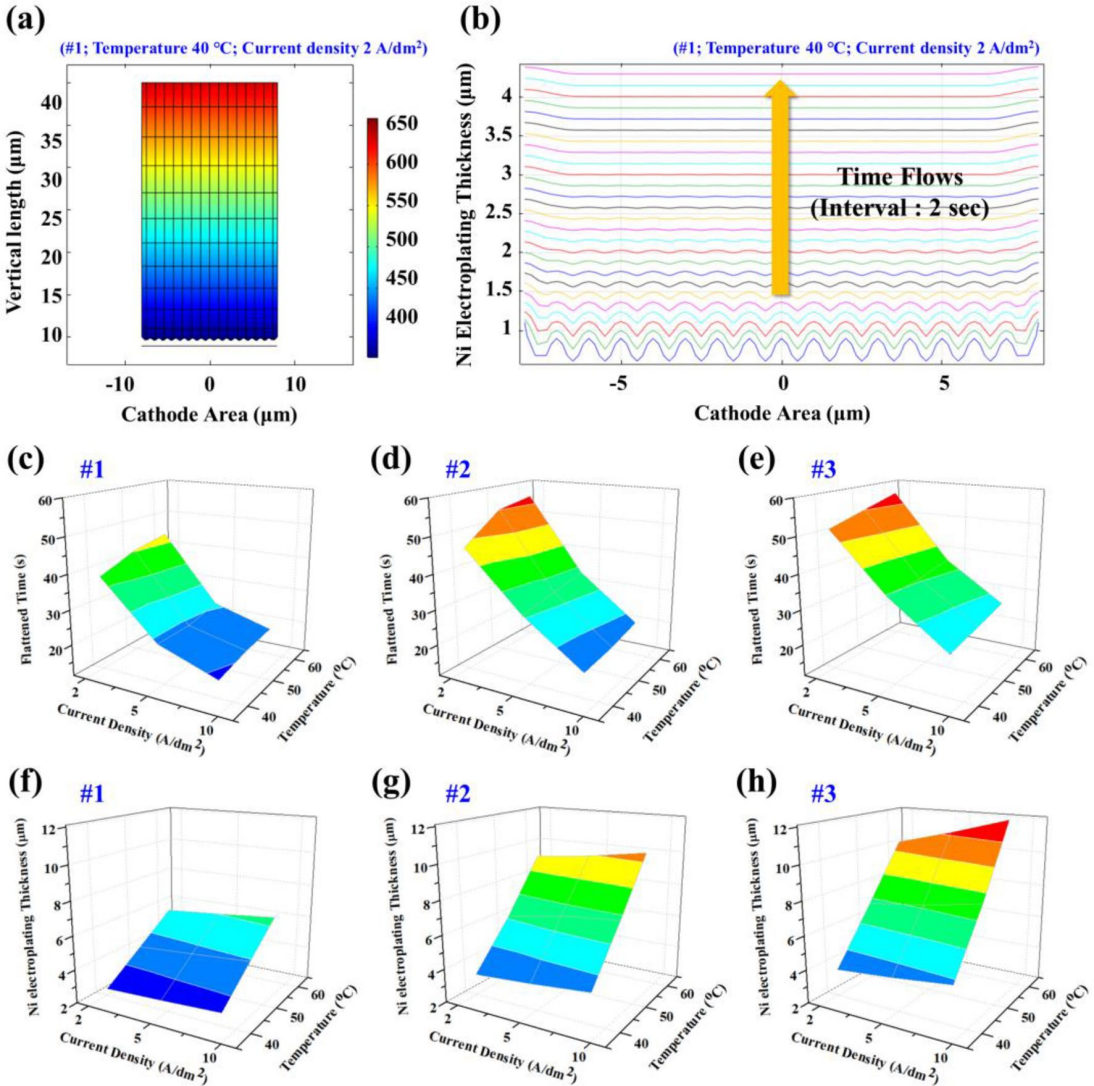
Multi-physics FEM simulation results. (**a**) the electrolyte potential contour image of #1 geometry, (**b**) the thickness of electroplated Ni layer with respect to time flows with an interval of 2 s. Time result when the cathode of (**c**) #1, (**d**) #2, (**e**) #3 geometry becomes flat under each current density and temperature condition. The thickness results of the electroplated cathode of (**f**) #1, (**g**) #2, (**h**) #3 geometry after a fixed time of 30 s.

The thickness and smoothness of the Ni layer for each condition was simulated and presented graphically as shown in Fig. 5a,b. In Fig. 5a, the electrolyte potential is visualized with a contour image at a given electroplating condition (#1; temperature, 40 °C; current density, 2 A/dm^2^). As the time progresses, the potential of the electrolyte gradually decreased and the Ni^+^ ions in the electrolyte were deposited on the cathode electrode. As shown in Fig. 5b, the roughness of the cathode gradually decreases as Ni is electroplated. For each of the 3 roughness types (#1, #2, #3) (Fig. 3b–d, Table 3), the simulation proceeded under the conditions of 3 current densities (2, 5, 10 A/dm^2^) and 3 temperatures (40, 50, 60 °C).

The flattened time for each condition is presented as 3D surface graphs (Fig. 5c–e, Table 6). As the surface roughness increased (#1 → #3), it was found that the time for the cathode to be flattened increased in all conditions. In addition, it was observed that the flattened time rapidly decreased as the current density increased (2 → 5 → 10 A/dm^2^). This is due to the faster transport of Ni ions in the electrolyte to the cathode surface at increasing current densities. Furthermore, it was found that temperature did not significantly affect the flattened time.

**Table 6:** Simulated flattened time of cathodes #1, #2, and #3 at various current densities and temperatures.

Time (s)		Temperature (°C)								
		#1			#2			#3		
		40	50	60	40	50	60	40	50	60
Current Density (A/dm^2^)	2	38	42	44	46	54	56	51	54	57
	5	22	22	24	30	32	34	35	36	38
	10	16	18	19	18	19	21	23	25	27

The thickness of the Ni electroplated layer is shown in a 3D graph after a constant period of 30 s (Fig. 5f–h, Table 7). It was found that as the surface roughness increased, the thickness of the plated Ni layer increased significantly. If the area to be plated with Ni decreases relatively quickly (smoothens), such as case #1, the plating reaction slows. In comparison, a rough surface such as case #3 does not achieve flattening in 30 s; due to this, case #3 retains its roughness and higher surface area for longer and sustains an accelerated Ni deposition process. In addition, temperature increase also increased the deposit thickness; the effect of temperature on thickness is contrasted with its limited effect in flattened time. Overall, an increase in the Ni deposit thickness indicated a growth in plated Ni particle size. As the size of the Ni particles increases, the voids between Cu particles can be quickly filled. However, if plating is allowed to continue after flattened time, excessive Ni growth will begin to increase overall roughness.

**Table 7:** Simulated final thickness of the electroplated cathodes #1, #2, and #3 after a fixed time of 30 s.

Thickness (μm)		Temperature (°C)								
		#1			#2			#3		
		40	50	60	40	50	60	40	50	60
Current Density (A/dm^2^)	2	2.40	2.55	2.73	3.25	3.50	3.83	3.6	3.9	4.3
	5	3.95	4.20	4.45	5.90	6.50	6.83	6.8	7.2	7.8
	10	5.50	5.80	6.15	9.10	9.50	10.1	10.1	11.0	12.0

Based on the simulations, it was demonstrated that the current density is related to the Ni electroplating speed, and the temperature is related to the size of the Ni particles. Therefore, after identifying these phenomena, experiments were performed to confirm simulation findings. As predicted based on the simulation results, if electroplating is performed under high current density and high temperature conditions, larger size particles are plated faster, and thus a flatter Ni layer can be formed in a shorter period.

Following FE simulation results, Ni electroplating experiments were conducted at several parameters. As shown in Table 8, various specimens were prepared with 3 independent electroplating variables: temperature, current density, and plating time. In addition, dependent variables: resistivity, surface roughness, thickness, and adhesion of the Ni layer were measured for the 18 specimen types.

**Table 8:** Experimental results of Ni electroplating parameters on: resistivity, surface roughness, thickness, and adhesion.

Expt. no	Factors			Resistivity (μΩ∙cm)	Surface Roughness (S_q_) (μm)	Thickness of Ni (μm)	Adhesion
	Bath temperature (°C)	Current density (A/dm^2^)	Time (min)				
S-1	40	2	1	38.20	3.90	2.22	3B
S-2	40	2	3	32.36	3.44	3.51	4B
S-3	40	2	5	21.96	3.29	3.82	5B
S-4	40	5	1	29.68	3.28	3.51	4B
S-5	40	5	3	19.01	2.67	5.01	2B
S-6	40	5	5	12.68	2.47	7.35	1B
S-7	40	10	1	32.34	1.67	5.74	0B
S-8	40	10	3	10.54	2.28	6.36	0B
S-9	40	10	5	8.964	2.74	6.55	0B
S-10	60	2	1	45.52	4.56	2.98	5B
S-11	60	2	3	38.01	4.07	4.79	5B
S-12	60	2	5	24.31	3.43	5.63	5B
S-13	60	5	1	30.63	3.57	5.67	5B
S-14	60	5	3	18.27	2.58	7.84	3B
S-15	60	5	5	10.44	2.19	11.46	1B
S-16	60	10	1	14.78	1.45	5.41	5B
S-17	60	10	3	14.23	2.29	10.45	1B
S-18	60	10	5	10.97	2.38	13.23	0B

The resistivity of bulk Ni is about 7 μΩ∙cm, but when electroplated, about 3 times the resistivity has been previously observed^34–37^. When electroplating Ni onto a CPN electrode with an initial resistivity of 12.2 μΩ∙cm, the electrodes final resistivity differed depending on the plating conditions. It was found that as the thickness of the Ni layer increased, the resistivity decreased and approached the initial resistivity of the CPN electrode.

In the cases of S-6, S-8, S-9, S-15, S-17 and S-18 with very thick Ni layer, the resistivity was lower than that of the initial CPN electrode. This is due to the Ni filling in the CPN electrode increasing electron pathways. However, the electroplating proceeded excessively inside of the Cu electrode, weakening and almost eliminating the adhesion between the electrode and the substrate. S-16 is the only case in which substrate adhesion was retained while maintaining a relatively low resistivity of 14.78 μΩ∙cm and a low surface roughness.

At a fixed plating time, the surface roughness, thickness, and adhesion of plated Ni all increased with increased temperatures. This is in-line with simulation results. Under the same conditions, when the temperature increased, the thickness of the Ni electroplated layer also increased (Fig. 5f–h). Additionally, increasing current density increased the thickness of the Ni deposit and formed a smoother surface. Furthermore, an increase in plating time led to an increase in thickness and a decrease in roughness.

Another phenomenon was observed by varying current density while fixing other independent variables. Increasing current density resulted in an increase of the plating’s final thickness, but it also resulted in an increase to the plating’s final roughness. At a higher current density of 10 A/dm^2^, the cracks on the surface of the CPN electrode had been quickly filled by large Ni particles. Continued growth of the Ni deposits led to a high surface roughness. Over longer periods of plating time, the surface roughness would continue to increase beyond the initial roughness of the CPN electrode. At a lower current density of 5A/dm^2^, it was observed that smaller Ni particles had gradually filled the cracks of the CPN electrodes surface and created a smooth Ni surface. The same trend occurred under all conditions, independent of temperature. In the case of S-16, electroplating was performed at a high temperature of 60 °C and a high current density of 10 A/dm^2^, resulting in relatively large diameter Ni particles being deposited on the electrodes surface at a high rate. Due to case S-16’s low plating time of 1 min, it was possible to fill the cracks in the CPN electrodes surface with large Ni particles while still maintaining a relatively low surface roughness. In the cases of S-17 and S-18, which had proceeded for more than 3 min, the Ni growth formed was excessively thick and as a result, the electrodes final roughness had drastically increased. In addition to the increased roughness, Ni had been deposited inside the electrode, leading to an inhibition of adhesion to occur between the CPN and the substrate. In summary, the S-16 specimen showed the best balance of surface roughness, plating thickness, and adhesion to the substrate.

Based on the superior performance of the S-16 Ni-electroplated CPN electrodes, further samples were fabricated with S-16 parameters. The S-16 Ni-electroplated CPN electrodes are compared with non-plated Cu NP electrodes and CPN electrodes. In addition, S-16 electroplating conditions were utilized for selective plating experiments. By using aluminum foil, half of the printed CPN electrode was masked then exposed to IPL sintering at the optimal 2800 V 3 ms on-time flash. During electroplating, alligator clips were placed at the middle junction of the two halves and immersed in the same electrolyte.

Achieving selective plating of only the sintered potion presents significant savings in circuit manufacturing time. Like negative photoresists, only the printed CPN surfaces exposed to IPL irradiation will accommodate Ni plating. This can be utilized to create re-usable patterned masks with the desired circuit pattern to be plated. Under S-16 conditions, Ni plating was not observed in the non-sintered portion of the electrode while the sintered half plated normally. As expected, the non-conductive portion is unable to accommodate the negative charge provided by the power supply potential. This lack of conductivity meant the non-sintered portion could not participate in the reduction of Ni cations onto its surface. After over 5 min of plating time at S-16 conditions, the non-sintered portion exhibited no Ni-deposition. Furthermore, the non-sintered portion lost adhesion to the substrate and coherency while in the electrolyte. Simple shaking was able to remove the non-sintered portion, leaving behind a robust selectively Ni-plated CPN electrode.

**Figure 6:**
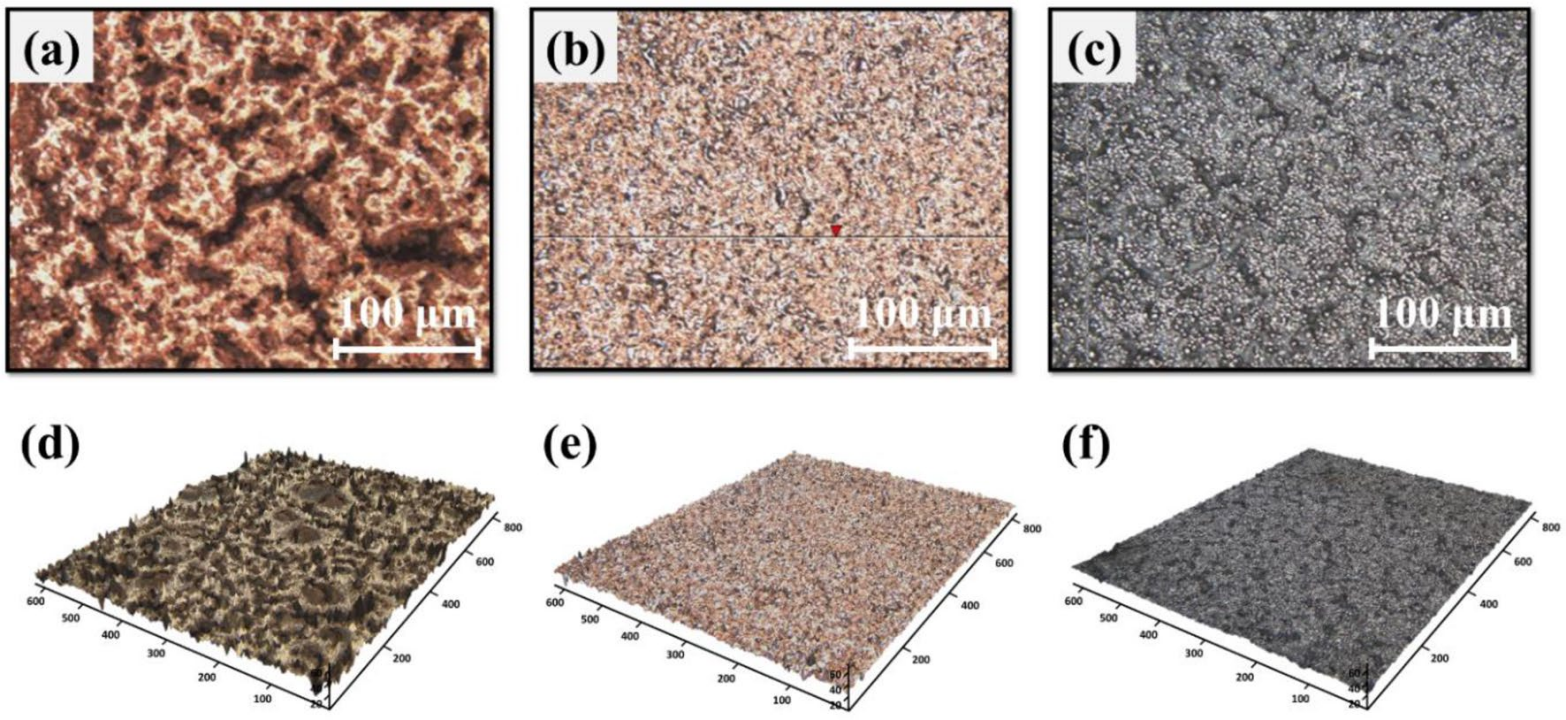
Surface morphology observations of (**a**) IPL sintered Cu NP electrode, (**b**) IPL sintered CPN electrode, and (**c**) Ni electroplated CPN electrode (S-16). Optical 3D profile images of an (**d**) IPL sintered Cu NP electrode, (**e**) IPL sintered CPN electrode, and (**f**) Ni electroplated CPN electrode (S-16).

Using a 3D optical profiler, the surface roughness of three kinds of electrodes were compared (Fig. 6). Pure Cu NP electrodes had a significantly higher surface roughness of *S*_*q*_ = 12.04 μm, as shown in Fig. 6a, and Table 9. Alternatively, the surface roughness of the CPN electrode was reduced to *S*_*q*_ = 3.90 μm as a result of Cu seeds that filled the voids between Cu particles. In addition, when Ni electroplating was performed on the CPN electrode, its surface roughness was drastically reduced to *S*_*q*_ = 1.45 μm.

**Table 9:** Surface characterization parameters of three kinds of electrodes (Observation Area: 831 μm × 623 μm).

Label	IPL sintered Cu NP electrode	IPL sintered CPN electrode	Ni-electroplated CPN electrode (S-16)
*S*_*q*_ (μm)	12.04 ± 2.01	3.90 ± 0.44	1.45 ± 0.31

**Figure 7:**
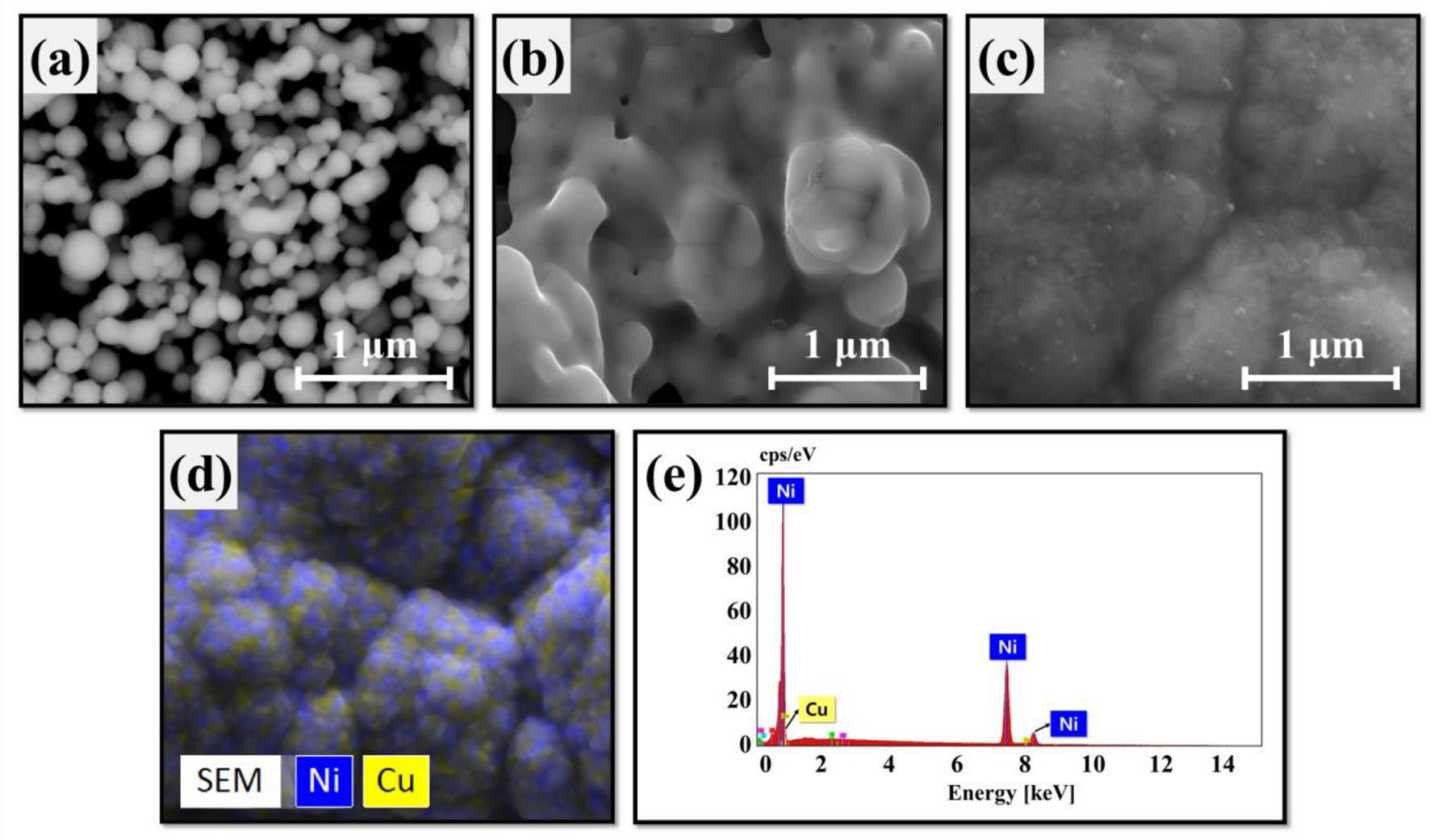
SEM analysis of an (**a**) IPL sintered Cu NPs electrode, (**b**) IPL sintered CPN electrode, and (**c**) Ni electroplated CPN electrode (S-16). (**d**) EDS mapping for a Ni electroplated CPN electrode and an (**e**) EDS spectrum for Ni electroplated CPN electrode (S-16).

The changes in roughness were observed in further detail through SEM analysis. Figure 7a shows the surface of IPL sintered Cu NPs. Although necking occurred between the particles, there were many voids and cracks still present between these particles. On the other hand, as seen in Fig. 7b, when Cu seeds are used as the precursor, the connections between Cu NPs are much larger, resulting in a more densely sintered material. Finally, in the case of Fig. 7c, it was observed that the Ni plated surface was smooth relative to the other electrodes.

EDS analysis was performed to confirm that Ni was uniformly deposited onto the surface of the CPN electrode. Figure 7d shows the EDS mapping for the distribution of Cu and Ni while Fig. 7e shows the corresponding EDS spectrum. On the EDS map, the blue Ni component is shown to cover the entire surface, with the yellow Cu component being detected in between the Ni layer. However, when this was examined by the EDS spectrum, the detected Cu peak was relatively negligible compared to the detected Ni peak. From this, the conclusion was drawn that the Ni layer covers the majority of the CPN electrode.

To demonstrate the densification of each electrode, SEM images were processed by adjusting the brightness of the images. Through the emphasized contrast between black and white, solids were represented by white regions and the voids were represented by black regions. The densification of the electrode was estimated by finding the ratio of the number of white pixels in the images to the total number of pixels in the image. 79.4% of the 336,788 total pixels were white in the processed Cu NP electrode image, while 98.6% of the 336,788 total pixels were white in the CPN electrode image. It was therefore concluded that the voids within the Cu NP electrodes were decreased from 20.6 to 1.4% when the Cu precursor was added. In the case of the Ni-plated CPN electrode, 100% of the 336,788 pixels in the image were white, indicating that the voids within the electrode had been filled in. It was demonstrated that voids present between Cu NPs could be largely eliminated by subjecting Ni electroplating to CPN electrodes.

**Figure 8:**
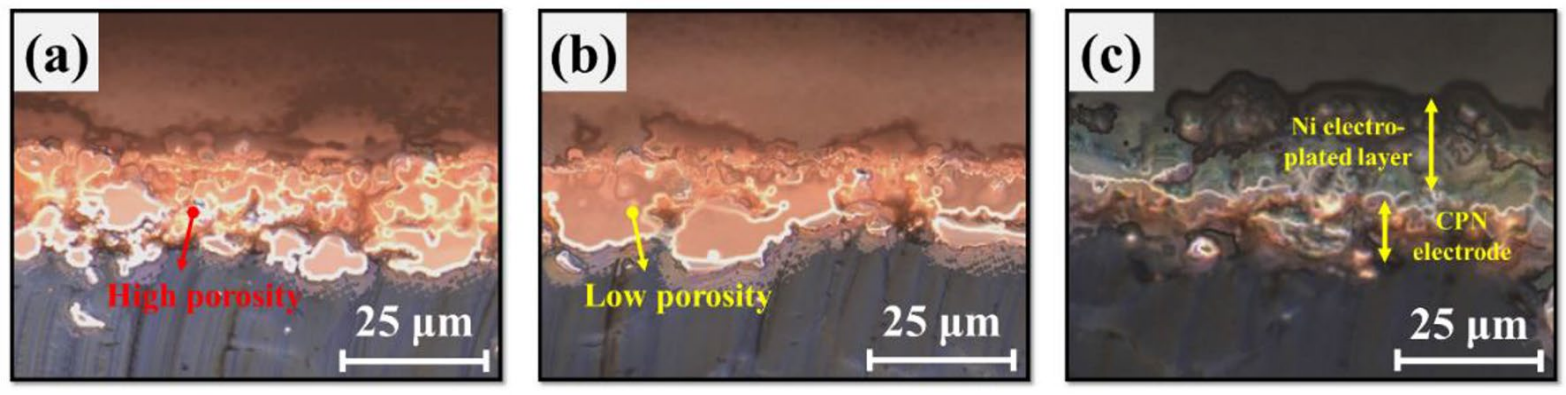
Cross-sectional observation of an (**a**) IPL sintered Cu NPs electrode, (**b**) IPL sintered CPN electrode, and (**c**) Ni electroplated CPN electrode (S-16).

These results were further confirmed through cross-sectional observation of three kinds of electrodes (Fig. 8). Each specimen was immersed in liquid nitrogen for 10 min and then split to expose their cross-sections and observe them through an optical microscope. The cross section of the Cu NP electrode revealed that the particles within were sintered, but many voids between the particles were still observed (Fig. 8a). In the case of the CPN electrode, its porosity was reduced due to the Cu seeds that filled the voids between Cu NPs, while also retaining a smooth surface (Fig. 8b). As shown in Fig. 8c, it was observed that the Ni plated CPN electrode was covered in a homogenous Ni layer. In addition, it was observed that the Ni layer penetrated the inside of the Ni plated CPN electrode and filled the fine cracks of the CPN electrode. Therefore, it can be inferred that the entire surface of Cu is covered with Ni, and a strong bonding force acts between the interfaces. Since a homogeneous layer of Ni metal has formed on the surface, the Ni plated CNP electrode was theorized to have better solder adhesion.

**Figure 9:**
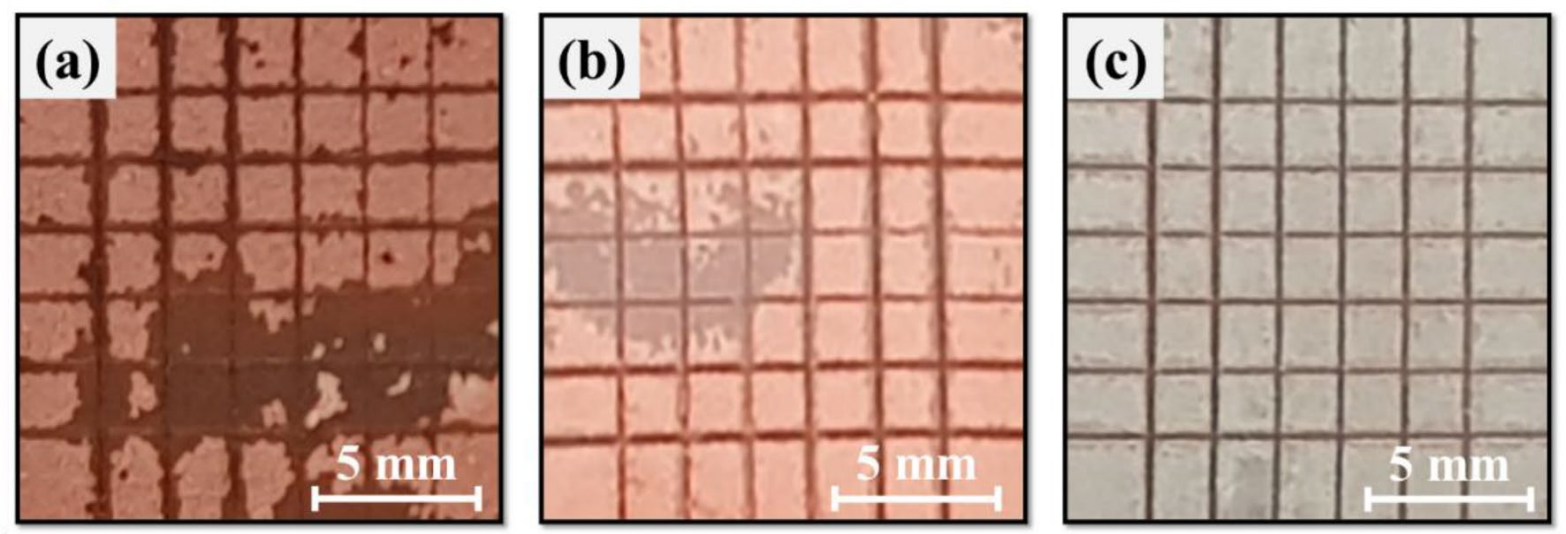
ASTM D 3359 Adhesion test for an (**a**) IPL sintered Cu NP electrode, (**b**) IPL sintered CPN electrode, and (**c**) Ni electroplated CPN electrode (S-16).

In order to perform as a solderable electrode, there must be a strong bonding force between the solder-Ni layer and the Ni layer-CPN electrode. Therefore, ASTM D3359 adhesion testing was performed on three electrodes: Cu NPs (Fig. 9a), CPN (Fig. 9b) and Ni-electroplated CPN (Fig. 9c). In the case of the Cu NP electrode, the solder-electrode bonding strength was too weak to form a viable connection due to its rough surface and high porosity. During the adhesion test, more than 35% of the grids surface area flaked off, indicating 2B adhesion. In the case of the CPN electrode, it was found that over 15% of the grids surface area gradually flaked off, indicating 3B adhesion. In the case of the Ni electroplated CPN electrode, the electrode completely maintained its Ni surface, indicating 5B adhesion. This confirmed that both the mechanical and surface morphology characteristics of Cu NP electrodes can be improved through Ni electroplating treatment.

### Wetting and Mechanical properties of the Ni-electroplated CPN electrode

**Figure 10:**
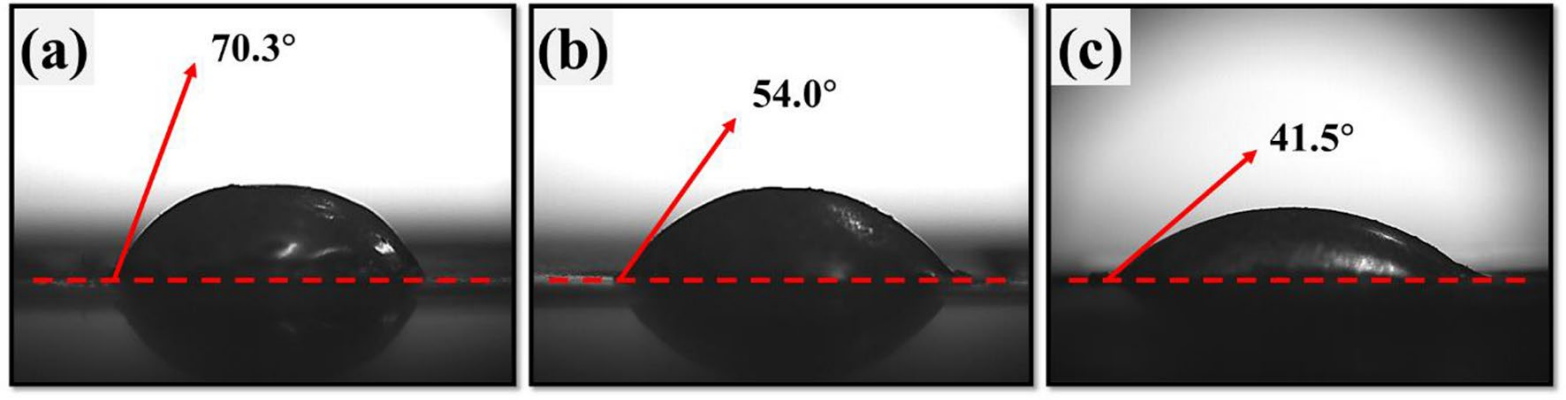
Solder contact angle measurement of an (**a**) IPL sintered Cu NPs electrode, (**b**) IPL sintered CPN electrode, and (**c**) Ni electroplated CPN electrode (S-16).

To investigate the solderability of Ni-electroplated CPN electrodes, wetting and solder shear tests were conducted. The wetting test was conducted by melting 1 cm of solder wire with a soldering iron, dropping the solder onto the electrode, and then measuring the contact angle between the solder and the electrode. As shown in Fig. 10a, the solder dropped onto the Cu NP electrode retained a relatively low-width elliptic shape resulting in a contact angle of 70.3° between the solder and the electrode. In comparison, the CPN electrode retained a contact angle of 54.0° between the solder and the electrode as shown on Fig. 10b. The Ni-electroplated CPN electrode exhibited the most solder-wet behaviour with an even further decreased contact angle of 41.5° between the solder and the electrode as shown in Fig. 10c. The decreased surface roughness and homogenous Ni surface was attributed for the improved solder wetting properties of the electroplated CPN electrode.

**Figure 11:**
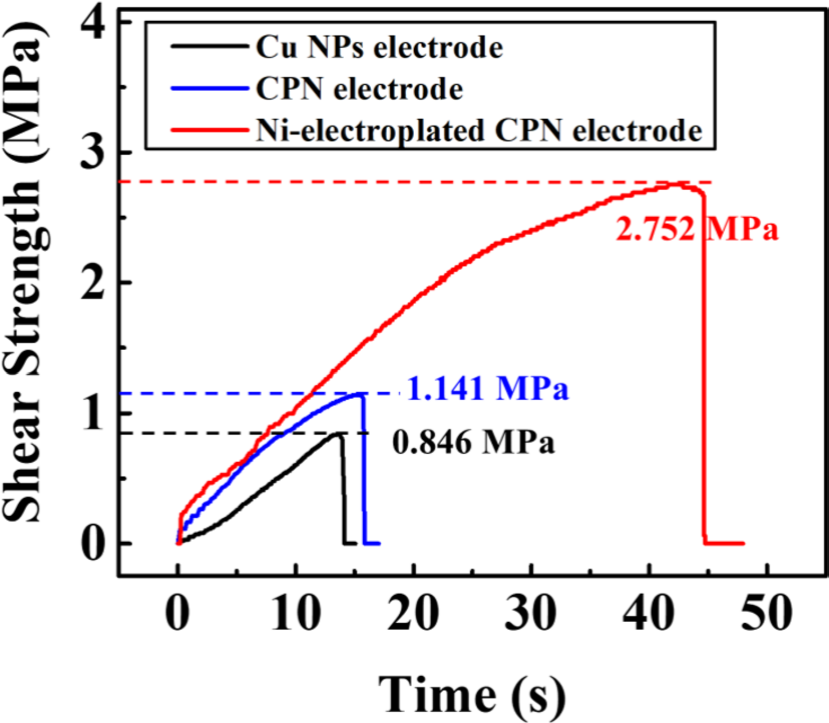
Shear strength tensile test between solder and electrodes. Ni-electroplating was performed under S-16 conditions.

Shear tensile testing was performed to quantitatively determine the bonding strength between solder and the electrode. Solder shear strength was measured using a tensile tester as shown in Fig. 11. Through this experiment, the relationship between the electrode’s treatment and the electrodes solder wettability was determined. The highest maximum shear strength of solder was observed on the Ni-electroplated CPN electrodes at 2.75 MPa. Based on these experimental results, it was confirmed that the mixing of Cu precursor and Ni electroplating significantly lowered the roughness of the electrode surface, and thus significantly increased its bonding strength with the solder. This is attributed to an increase in the electrodes solder wetting properties, resulting in a higher contact area and thus, a stronger solder connection.

Through the present investigations, sintering and electroplating parameters were optimized at 2800 V and S-16 conditions, respectively. IPL sintering at 2800 V provided the necessary energy required to sufficiently sinter the printed electrodes while avoiding excessive oxidation at higher energies. The addition of Cu precursor was found to improve conductivity by seeding Cu and increasing the overall packing density and electron pathway. Electroplating at higher temperatures (60 °C) and current density (10 A/dm^2^) facilitated rapid void filling. Limiting the plating time to 1 min avoided excessive Ni growth which would increase surface roughness at longer electroplating times. Selective electroplating was demonstrated by utilizing a simple mask during IPL sintering to create conductive and electroplating surfaces while rejecting non-sintered portions (Fig. 2).

By utilizing Cu precursors and electroplating: solderability, adhesion, and mechanical strength was improved to produce a robust and conductive electrode. An important detail to note is that the Ni electroplated CPN electrode shearing test did not fail between the solder and the Ni surface, nor between the Ni surface and the CPN electrode. In fact, the shear test failed between the CPN electrode and the PI substrate. From this observation, it was concluded that the solderability was maximized for this type of electrode without further improvements to adhesion between the electrode and the substrate. Although chosen for its mechanical and electrical properties, there are other alternative plating metals instead of Ni. Future works can be performed to explore alternative plating metals to reduce cost while retaining the mechanical and conductive properties of Ni plated CPN electrodes.

## Conclusions

We investigated the feasibility of nickel plating on IPL sintered hybrid copper electrodes. The analytical and experimental tests were conducted to observe the morphology characteristics of different electrodes to improve the solderability of printed Cu electrodes. Cu NPs were mixed with 30 wt.% of Cu (II) Nitrate Trihydrate precursor to fabricate the Cu Precursor/NPs (CPN) electrodes. It was found that the resistivity of the CPN electrodes was decreased due to the reduction and precipitation of Cu precursor between Cu NPs. Ni electroplating process was applied to complement the weak mechanical properties of IPL sintered Cu, which further improved the surface morphology characteristics and improved solderability. Through multi-physics FEM electroplating simulations, effects of different parameters on electroplating were identified. Based on the mechanistic insight provided by simulation results, Ni-electroplating was performed for improved solderability and mechanical properties. The surface roughness was drastically reduced to *S*_*q*_ = 1.45 μm in optimized conditions (temperature, 60 °C; cathode current density, 10 A/dm^2^; plating time, 1 min). Experimental solder shear tensile test showed a shear strength of Ni-electroplated Cu NPs/Precursor electrodes as 2.75 MPa. Reducing the roughness on a surface through Ni-electroplating leads to improved solder wetting. Moreover by applying a mask during IPL sintering, the electrode showed selective nickel plating only on the sintered portion (i.e., the conductive region); this paves the new way for patterned mask flashing followed by selective electroplating to streamline the circuit manufacturing process. It is expected that the proposed approach can also be used in reflow soldering of electrical components for IPL based printed electrodes and circuits.
